# Mediating effects of burden on quality of life for caregivers of first-time stroke patients discharged from the hospital within one year

**DOI:** 10.1186/s12883-018-1057-9

**Published:** 2018-04-25

**Authors:** Yu-Hsia Tsai, Meei-Fang Lou, Tsui-Hsia Feng, Tsung-Lan Chu, Ying-Jen Chen, Hsueh-Erh Liu

**Affiliations:** 1 School of Nursing, College of Medicine, Chang Gung University, No. 259, Wenhwa 1st Road, Guishan Dist., Taoyuan City, 33302 Taiwan; 2 School of Nursing, College of Medicine, National Taiwan University, No. 1, Sec 1, Jen-Ai Rd, Taipei, 10051 Taiwan; 3 Department of Cardiovascular Medicine, Linkou Chang Gung Memorial Hospital, No.5, Fuxing St., Guishan Dist., Taoyuan City, 33305 Taiwan; 4 Administration Center of Quality Management Department, Taoyuan Chang Gung Memorial Hospital, No.123, Dinghu Rd., Guishan Dist, Taoyuan City, 33378 Taiwan; 5 Division of General Internal Medicine and Geriatrics, Department of Internal Medicine, Linkou Chang Gung Memorial Hospital, No.5, Fuxing St., Guishan Dist., Taoyuan City, 33305 Taiwan; 6 College of Medicine, Chang Gung University, No. 259 Wenhwa 1st Road, Guishan Dist., Taoyuan City, 33302 Taiwan; 7 Department of Rheumatology, LinKou Chang Gung Memorial Hospital, No.5, Fuxing St., Guishan Dist., Taoyuan City, 33305 Taiwan; 8 Department of Nursing, College of Nursing, Chang Gung University of Science and Technology, No. 261, Wenhua 1st Rd., Guishan Dist., Taoyuan City, 33303 Taiwan

**Keywords:** Burden, Quality of life, Mediating effect, Caregiver, Stroke

## Abstract

**Background:**

Caregiver burden may be either a predictor or an outcome of caregiver quality of life (QoL). Patient or caregiver factors that directly affect caregiver QoL, predictors that are simultaneously shared with caregiver burden and QoL, and factors that affect caregiver QoL through caregiver burden are not well understood. This study explored predictors of caregiver QoL and identified whether caregiver burden is a mediator for caregivers of first-time stroke patients.

**Methods:**

This is a cross-sectional study. We recruited first-time stroke patients who had been discharged from the hospital within 1 year. We screened caregivers with two major inclusion criteria: age > 20 years old and being the family member who provides the most patient-care hours out of all family caregivers. Caregiver burden (Caregiver Strain Index, CSI), QoL (Caregiver Quality of Life Index, CQLI), and patient and caregiver characteristics were assessed with structured questionnaires. Multiple-regression and bootstrap analysis were conducted for data analysis.

**Results:**

A total of 126 caregivers completed the questionnaires. Higher caregiver burdens, lower caregiver education level, lower self-rated health, lower monthly family income, and spouses who were responsible for medical fees were significant predictors of lower caregiver QoL. Poor self-rated health and monthly family income of $ 666 USD or below were the strongest predictors of caregiver QoL. Spouses who were responsible for medical fees and lower monthly family income had direct negative effects on caregiver QoL, but these factors exhibited no indirect mediating effect between caregiver characteristics and QoL through caregiver burden as a mediator. Caregiver education level at or below elementary school and poor or fair self-rated-health had direct negative effects on caregiver QoL, which were mediated by caregiver burden.

**Conclusions:**

Our study indicated predictors of caregiver QoL and the relationships with caregiver burden among first-time stroke survivors in the early stage. Caregivers’ financial factors affected caregiver QoL directly. Caregivers’ poor self-rated health and lower education level negatively affected caregiver QoL indirectly through caregiver burden as a mediator. Interventions to make appropriate policies for financial subsidies, to enhance caregivers’ health and to provide tailored stroke-related education through multidisciplinary cooperation may effectively promote caregiver QoL.

## Background

Most stroke survivors rely on family caregivers to assist in managing stroke-related deficits and reintegration into the community [1, 2]. Numerous studies have been conducted to explore the quality of life (QoL) of the family caregivers of stroke patients during various stages of treatment or rehabilitation in different countries [3–6]. Additionally, both positive and negative impacts of stroke survivors on caregivers have been explored through cross-sectional and longitudinal studies [7, 8]*.*

The relationships between characteristics of the stroke patient or the caregiver, caregiver burden, and caregiver QoL appear related to others, yet the direction of these relationships remains unclear among various populations. Several studies have shown that caregiver burden is either a strong determinant of caregiver QoL [9–11] or an outcome instead of a predictor [12]. As such, caregiver burden could have a mediating effect between the patient or caregiver characteristics and the caregiver's QoL. Mediation analysis explores the process underlying how one variable influences another variable [13]. Caregiver burden with a mediating effect is consistent with limited earlier studies [3, 14]. Caregiver characteristics (spouse, caregiver's health status) and family resources (family income) were identified as significant predictors of caregiver QoL with a mediating effect of caregiver burden among stroke survivors receiving rehabilitation therapy [3]. The association between the characteristics of both patients and caregivers, as well as the caregiver's burden and QoL, revealed various results in different stages of stroke patients undergoing rehabilitation [14]. The caregiver's burden and QoL were associated with patient and caregiver characteristics (age, gender or mood), as well as family resources (family networks). Of these variables, only patient and caregiver characteristics affected caregivers' burden and QoL at the early stage of post-stroke. Furthermore, patient dependency and family resources were additional independent predictors for caregivers' well-being at a later stage [14]. However, only a limited number of studies have investigated QoL and associated factors affecting family caregivers for first-time stroke survivors within the critical one-year period of home care after being discharged from the hospital. Understanding the association between caregivers' QoL and influencing factors could be beneficial for mediating caregiver burden and improving caregiver QoL. The results from these investigations will help health providers develop caregiver interventions for post-stroke patients from the early stage, especially for first-time stroke families.

We hypothesized that there is an association among variables related to first-time stroke patients and caregivers, family resources, and caregiver QoL, and this relationship can be mediated by reported caregiver burden. Thus, to determine which patient or caregiver factors directly affect caregiver QoL, which predictors are simultaneously shared with caregiver burden and QoL, and which factors affect caregiver QoL through caregiver burden, the aim of this study was to explore the predictors of caregiver QoL by testing this hypothetical relationship and determining whether caregiver burden was a mediator for the QoL of caregivers of first-time stroke patients discharged from the hospital less than a year previously.

## Methods

This was a cross-sectional study. We recruited subjects from lists of first-time stroke patients who had been discharged within 1 year. These lists were provided by a medical center, three regional hospitals, and a local registration system in a public health center located in northern Taiwan. We initially screened caregivers by phone for the following inclusion criteria: age > 20 years old and being the major family caregiver who provided the most hours in taking care of stroke patients. Caregivers could either be full- or part-time and were able to communicate verbally. After ethical approval was obtained from the Research Ethics Committee of Medical Foundation Institutional Review Board (No. 95-0489B), phone calls were made to individuals on the list to explain the purpose of the research, request participation, and screen for potential caregivers. Oral agreement was obtained before a home visit, and written informed consent was obtained from all participants before data collection. Participants completed questionnaires by face-to-face interview at caregivers’ homes. A total of 184 families agreed to participate; however, only 126 primary caregivers fit the selected criteria and completed questionnaires.

The independent variables were patient characteristics, caregiver characteristics, and family resources. The characteristics of stroke survivors consisted of demographic data and health information, including age, gender, marital and employment status, past medical history, duration of hospitalization, use of tracheostomy tubes, nasogastric (NG) tubes, Foley catheters, assistive devices for walking, activities of daily living (ADL) (Bartel Index, BI), and cognitive status (Short Portable Mental Status Questionnaire, SPMSQ) at the time of data collection. The BI is a widely used measure of ADL. The range of scores is 0-100, with a lower score indicating a higher dependent status of the subject [15]. Cognitive status was measured by the Chinese version of the SPMSQ [16], which has good reliability [17]. The total number of correct answers was adjusted by the educational level of subjects to produce the SPMSQ score (range 0-10). Caregiver characteristics consisted of demographic data and health information, including age, gender, education, employment and marital status, living with other family members, and self-rated health status. Additionally, family resources included monthly family income, the identity of the person responsible for the majority of the patient’s medical fees, the number of family members living together, the number of family members helping with caregiving tasks, and the presence of a hired non-family caregiver.

Another independent variable was the Caregiver Strain Index (CSI) total score. CSI was selected to measure the caregiver burden in five domains: employment, financial, physical, social and time stress [18]. The index consists of 13 items with yes (score = 1) / no (score = 0) answers for a total score range of 0-13, with a higher score indicating a higher caregiver burden. The internal consistency of the CSI Chinese version was good [19], and the reliability coefficient of Kuder-Richardson Formula 20 in this study was 0.93. In our study, the total CSI score was treated as a mediating variable between caregivers’ characteristics and their QoL.

The dependent variable was the total score of the Chinese version of the Caregiver Quality of Life Index (CQLI), which was chosen to measure the caregiver QoL. This index was developed by McMillan and Mahon [20], with four items on a 0-to-100-mm visual analogue scale to measure the emotional, social, financial, and physical aspects of QoL. Several studies revealed that the CQLI had good internal consistency [21–23]. In this study, Cronbach's alpha was 0.75.

IBM SPSS Statistics version 23 was used for data analysis. Kolmogorov-Smirnov test results showed that the variables were normally distributed (*p* > 0.05). Univariate analyses (t-test, ANOVA and Pearson’s correlation) and multiple-regression analyses were conducted to identify predictors of caregiver QoL. We calculated power from the primary outcome of the multiple-regression. With our sample size of 126, power = 0.99 was computed under the effect size of 0.59, which meant our study achieved a large effect size [24], and our sample size was more than enough. All significance levels were set to 0.05 (2-tailed tests). To test whether caregiver burden had a mediating effect on caregiver QoL, a simple mediation model (one mediator existed) in the PROCESS SPSS computational tool was used. PROCESS is a modeling tool for SPSS and SAS that integrates many of the functions of statistical tools for mediation and moderation analysis [25]. Here, CQLI was treated as the outcome variable, and CSI was a mediator variable, while the other predictors were used as independent variables or covariates. Bootstrap analysis with 5,000 bootstrap samples was used to identify indirect effects. A 95% confidence interval (CI) was used to test the indirect effects of independent variables on the dependent variable via the mediator (caregiver burden, CSI). Mediation was considered significant if the bootstrapped 95% CI between the upper limit (BootULCI) and lower limit (BootLLCI) did not contain zero [26].

## Results

### Caregiver burden and quality of life

The median total CSI score was 6.5 (range: 0-13), and the median total CQLI score was 260.0 (range: 110-400). The lowest score was in the financial domain (median = 60.0; range: 0-100); whereas the highest score was in the emotional domain (median = 70.0; range: 20-100). The subscales in the physical (median = 70.0; range: 10-100) and social domains (median = 70.0; range: 30-100) had similar scores. Additionally, the higher the reported caregiver burden (total CSI score), the lower the caregiver QoL (total CQLI score) (r = -0.27, *p* = 0.002) (Table 1).

**Table 1:** Univariate analysis of the relationships between significant variables and CQLI total score

Variables	n (%)	CQLI Mean (SD)	r / t^a^/ F^b^	*p*	Comparison
Patient characteristics					
Using devices					
No	77 (61.1)	263.6 (54.7)	2.12^a^	0.036	No > Yes
Yes	49 (38.9)	242.7 (52.7)	–	–	–
Nasogastric tube					
No	121 (96.0)	258.5 (52.4)	3.17^a^	0.002	No > Yes
Yes	5 (4.0)	182.0 (62.2)	–	–	–
Tracheostomy tube					
No	118 (93.7)	258.8 (53.0)	2.70^a^	0.008	No > Yes
Yes	8 (6.3)	206.3 (58.3)	–	–	–
ADL (BI)	–	–	0.30	0.001	–
Cognitive status (SPMSQ)	–	–	0.19	0.037	–
Caregiver characteristics					
CSI	–	–	-0.27	0.002	–
Education					
< Elementary	34 (27.0)	234.4 (53.1)	4.48^b^	0.015	High school and College > Elementary
High school	66 (52.4)	258.9 (51.2)	–	–	
> College	26 (20.6)	274.0 (58.3)	–	–	
Self-rated health					
Poor	6 (4.8)	181.7 (39.7)	13.45^b^	< 0.001	Good > Fair
Fair	65 (51.6)	243.9 (47.8)	–	–	Fair > Poor
Good	55 (3.7)	277.1 (53.1)	–	–	Good > Poor
Variables	n (%)	CQLI Mean (SD)	F^b^/ t	*p*	Comparison
Family resources					
Monthly family income ($ NTD)^c^					
< 20,000	13 (10.3)	209.2 (49.7)	9.48^b^	< 0.001	60,000 >
20,000–60,000	55 (43.7)	247.5 (51.8)	–	–	20,000–60,000 >
> 60,000	58 (46.0)	273.4 (51.1)	–	–	20,000
Major payer for medical fees					
Self	46 (36.5)	263.4 (50.8)	2.74^b^	0.046	Self-paid and Children > Spouse and Other
Children	66 (52.4)	257.6 (57.1)	–	–	
Spouse	10 (7.9)	227.0 (44.7)	–	–	
Other	4 (3.2)	200.0 (34.6)	–	–	
Hire non-family caregiver					
No	103 (81.7)	260.7 (51.4)	2.34	0.021	No > Yes
Yes	23 (18.3)	231.7 (63.2)	–	–	–

Note. *CQLI* Caregiver Quality of Life Index, *ADL* activities of daily living, *BI* Barthel Index, *SPMSQ* Short Portable Mental Status Questionnaire, *CSI* Caregiver Strain Index, *SD* standard division, *NTD* New Taiwan Dollar

^a^ = t value; ^b^ = F value

(^c^: $ 20,000 NTD = $ 666 USD, $ 60,000 NTD = $ 2,000 USD)

### Patient characteristics and their association with caregiver quality of life

Most of the patients were elderly (66.1 ± 12.7 years; range: 35-97 years), male (60.3%), married (80.2%), and unemployed (61.1%). Most patients had no history of surgery (92.1%) or rehabilitation (65.1%); did not use assistive devices (61.1%), NG tubes (96%), or Foley catheters (96%); and had not undergone a tracheostomy (93.7%). The mean time since the stroke was 154.8 days (SD = 88.8; range: 4-315 days); the mean duration of hospitalization was 13.3 days (SD = 13.7; range: 2-90); the mean ADL was 81.2 (SD = 31.8; range: 0-100); and the mean cognitive status was 8.3 (SD = 3.1; range: 0-10). Results of the univariate analyses showed that five patient characteristics were significantly positively associated with caregiver QoL (Table 1): caring for patients without assistive devices (t = 2.12, *p* = 0.036), NG tubes (t = 3.17, *p* = 0.002) or tracheostomy tube (t = 2.70, *p* = 0.008), and caring for patients with lower dependent status (ADL, r = 0.30, *p* = 0.001) and better cognitive function (SPMSQ, r = 0.19, *p* = 0.037).

### Caregiver characteristics and their association with caregiver quality of life

Most of the caregivers were female (67.5%), high-school educated (52.4%), married (82.5%), patient’s children (44.4%) or spouse (42.1%), and were living with other family members (83.3%); in addition, most of the caregivers rated their health as fair (51.6%). The percentage of caregivers who were unemployed and employed was similar (50.8 versus 49.0%, respectively). Additionally, the mean caregiver age was 49.0 years (SD = 13.2, range: 20-81 years). Results of the univariate analyses revealed that caregivers with more education reported higher total CQLI score (*F* = 4.48, *p* = 0.015). The post hoc comparison revealed that caregivers with high school and college or above education had better QoL than those with elementary school or below education. Caregivers with better self-rated health reported better QoL in general (*F* = 13.45, *p* < 0.001). Further comparison showed that subjects with good health reported better QoL than those with fair or poor health, and subjects with fair health had better QoL than subjects with poor self-rated health status (Table 1). No significant relationships were found between QoL and gender (*t* = -1.195, *p* = 0.235) or age (*r* = -0.16, *p* = 0.074).

### Family resources and their association with caregiver quality of life

Family resources were characterized as monthly family income over $ 60,000 NTD (New Taiwan Dollar) ($ 2,000 USD) (46.0%), patients’ medical fees paid by their children (52.4%), sharing caregiving tasks (81.0%), and not hiring non-family caregivers (81.7%). Additionally, the average number of cohabiting family members was 4.2 (SD = 2.7, range: 0-22). Univariate analyses revealed that caregivers with a higher monthly family income reported higher total CQLI score (*F* = 9.48, *p* <  0.001). The post hoc comparison revealed that subjects with over $ 60,000 NTD ($ 2,000 USD) reported better CQLI than those with a monthly income of $ 20,000-60,000 NTD; and subjects with a monthly income of $ 20,000-60,000 NTD ($ 666-2,000 USD) reported better CQLI than an income of less than $ 20, 000 NTD ($ 666 USD). Self-paid caregivers, or if children paid for the medical expenditure, had better CQLI than when the spouse and others paid (*F* = 2.74, *p* = 0.046). Families that did not hire non-family caregivers reported better CQLI than those who hired additional helpers (t = 2.34, *p* = 0.021) (Table 1).

### Predictors of caregiver quality of life

Multiple-regression analysis revealed that caregivers with poor or fair self-rated health, elementary school or below education, monthly family income less than $ 20,000 NTD ($ 666 USD) or $ 20,000-60,000 NTD ($ 666-2,000 USD), medical fees paid by spouse and higher caregiver burden were significant predictors of lower caregiver QoL. These factors explained 37.4% of the total variance in the regression model (Table 2).

**Table 2:** Summary of regression analyses for caregiver quality of life

Variables	Caregiver quality of life (CQLI)		
	R^2^ change	βcoefficient	p*
Intercept		314.50	
Caregiver characteristic			
Self-rated health_ poor^b^	0.092	-57.36	0.007
Self-rated_ fair^b^	0.088	-22.88	0.009
Education < elementary^c^	0.026	-21.46	0.028
Family resource			
Monthly family income_ < $ 20,000 NTD^d,a^	0.046	-52.67	< 0.001
Monthly family income_ $ 20,000-60,000 NTD^d,a^	0.053	-25.25	0.004
Major payer of medical fees_ spouse^e^	0.027	-34.06	0.023
Caregiver burden			
CSI	0.043	-3.30	< 0.001
R^2^	0.374		

Note. *CQLI* Caregiver Quality of Life Index, *NTD* New Taiwan Dollar (^a^: $ 20,000 NTD = $ 666 USD, $ 20,000-60,000 NTD = $ 666-2,000 USD)

CSI = Caregiver Strain Index; * = p value for β coefficient

^b^ = referent category: caregiver self-rated health_good

^c^ = referent category: caregiver education > college

^d^ = referent category: monthly family income > $ 60,000 NTD ($ 2,000 USD)

^e^ = referent category: major payer of medical fees_self-paid

### The mediating effect of caregiver burden on caregiver quality of life

**Fig. 1:**
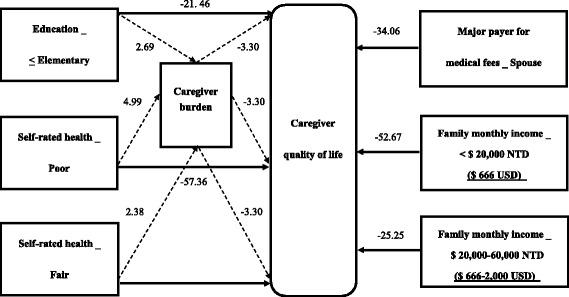
Predictors of caregiver quality of life. Legend:  direct effect;  indirect effect with caregiver burden as a mediator; NTD: New Taiwan Dollar

Figure 1 shows the indirect and direct effects of the mediation model with caregiver burden as a mediator of caregiver QoL. The results of mediating effects are shown in Table 3. Of the indirect effects, the bootstrapped 95% CIs of three variables (caregiver education no higher than elementary, and poor or fair self-rated health) do not contain zero; therefore, the results revealed significant indirect effects on caregiver QoL through caregiver burden as a mediator. The highest absolute value of β coefficients arose from the variable of poor self-rated health. The bootstrapped 95% CIs of the three other variables (spouse as the major payer for medical fees, monthly family income less than $ 20,000 NTD [$ 666 USD] or between $ 20,000 to 60,000 NTD [$ 666-2,000 USD]) contained zero in the indirect effects; therefore, these three variables exhibited no indirect mediating effect through caregiver burden as a mediator. Of the direct effects, the two variables with the highest absolute value of β coefficients were poor self-rated health and family monthly income less than $ 20,000 NTD ($ 666 USD), among six predictors of poor caregiver QoL.

**Table 3:** Summary of mediation effects on caregiver quality of life

Variables	Indirect effect		Direct effect		CSI as a mediator
	β coefficient (SE)	95% CI for Bootstrap [Lower, Upper]	β coefficient (SE)	95% CI for Bootstrap [Lower, Upper]	
Caregiver education < elementary	-8.88 (4.26)	[-2.52, -19.63]^b^	-21.46(9.65)	[-40.56, -2.35]^b^	Yes
Caregiver self-rated health_poor	-16.47 (9.12)	[-39.74, -2.72]^b^	-57.36 (20.09)	[-98.74, -15.97]^b^	Yes
Caregiver self-rated health_fair	-7.85 (3.33)	[-16.08, -2.66]^b^	-22.88 (8.58)	[-39.87, -5.89]^b^	Yes
Major payer of medical fees_ spouse	-0.87 (5.08)	[-12.60, 8.12]	-34.06 (14.73)	[-63.24, -4.89]^b^	No
Monthly family income < $ 20,000 NTD^a^	4.25 (5.79)	[-5.70, 17.62]	-52.67 (14.43)	[-81.25, -24.10]^b^	No
Monthly family income_$ 20,000-60,000 NTD^a^	4.54 (3.22)	[-0.45, 12.54]	-25.25 (8.53)	[-42.14, -8.37]^b^	No

Note. *CSI* caregiver strain index, *NTD* New Taiwan Dollar (^a^: $ 20,000 NTD = $ 666 USD, $ 20,000-60,000 NTD = $ 666-2,000 USD)

β coefficient effect of variable on caregiver quality of life, *SE* standard error, *CI* confidence interval for mediation test

^b^significant for direct or indirect effect, if the interval between the upper limit and lower limit of a bootstrapped 95% CI do not contain zero

## Discussion

### The associated factors and predictors of caregiver quality of life

This study revealed that most caregivers of first-time stroke survivors discharged from the hospital less than a year had moderate QoL. Caregiver characteristics including caregiver burden, and family resources were significant predictors of caregiver QoL among our three dimensions of patient or caregiver characteristics and family resources.

#### Patient characteristics

Our findings and previous research supported that stroke survivors’ poor health status, impaired cognitive function and ADL dependency play influential roles on caregiver QoL [7, 11]*.* However, these patient characteristics did not become a determining predictor of caregiver QoL in our research. The reasons may be that on average, the physical, cognitive and health status of our stroke survivors was not very severe, or compared to caregiver characteristics and family resources factors, patient characteristics might not be the most important factor among most of the our stroke survivors at the early stage of long-term care.

#### Caregiver characteristics

Consistent with other research, our study found that the following factors could predict poor QoL: poor or fair self-rated health and elementary school or below education [3, 6, 27]. In our study, poor caregiver self-rated health status was the most influential predictor of caregiver QoL, which was confirmed as a highly significant contributor to caregiver strain in previous research [5, 28]. Moreover, caregivers of first-time stroke survivors reported that they suffered from back pain, poor sleep quality, panic attacks, and feeling tired and frustrated [5, 29]. Consequently, both physical and mental strains could significantly affect caregiver QoL. Regarding caregivers’ educational issues, caregivers with higher education reported a better understanding of stroke-related disability and its consequences [28], provided more effective medical care and rehabilitation, had better coping strategies, and adapted better to their new roles as caregivers [6, 29]. Thus, lower education level might significantly result in lower caregiver QoL.

#### Family resources

Our study had similar results to another study [3] where family income and the spouse paying were predictors of caregiver QoL. Lower monthly family income had a negative effect on caregiver QoL [3], especially when considering that caregivers with a monthly family income of $ 20,000 NTD ($ USD 666) or below were the second strongest predictor in our study, behind only the predictor of caregivers with poor self-related health. Home caregivers of first-time stroke patients faced severe financial crises. These caregivers worried about financial strain and often quit their jobs to fulfil the role of caregiver [30]. Thus, home caregivers with lower family income would be a determining factor for lower caregiver QoL. Additionally, most stroke patients were cared for by aging spouses who had to endure the day-to-day demands of playing multiple roles at the individual, family and social levels [31]. These caregivers experienced more financial strain and negative impacts on their well-being than non-spouse caregivers [3]. These stressors might significantly and negatively affect spouse caregivers’ QoL, especially when they were responsible for the medical expenses.

Another interesting result was that compared to those who did not hire a non-family caregiver, caregivers who hired a non-family caregiver reported lower overall caregiver QoL. The need for additional help from others indicated that a caregiver might be overwhelmed by caregiving tasks. In our samples, ADL score among patients with “no hired non-family caregiver” (BI = 91.2 ± 21.6) was significantly higher than among those in the “hired” group (BI = 36.5 ± 31.7) (*t* = 7.9, *p* <  0.001). Hiring an additional helper might reduce the strains of caregiving. However, contradictory results were found in our study and another study [32]. Another helper necessitates increased communication between family members and the additional cost could exacerbate the economic and life strains on caregivers [32].

### Mediating effects on caregiver quality of life

#### Caregiver burden as a predictor and mediator of caregiver quality of life

Higher caregiver burden was an essential predictor of poor caregiver QoL in our study and other studies [3, 33]. Furthermore, we hypothesized that caregivers’ burden and their QoL might share similar predictors, and some predictors may affect caregiver QoL through caregiver burden as a mediator. Our study indicated that a caregiver with education no higher than elementary was a predictor of both higher caregiver burden and lower caregiver QoL. This factor of lower education level also indirectly affected caregiver QoL through caregiver burden as a mediator. Lower education might be associated with less ability to understand the process of caring for a first-time stroke patient and decrease the adaptability of the roles as a caregiver [29, 34]. Therefore, lower education may contribute to higher caregiver burden as well as to a lower caregiver QoL. Another mediating effect arose in the factor of caregiver health status, which was similar to another study [3]. In our study, “poor” self-rated health was the most important predictor for both the poor caregiver QoL and higher caregiver burden. This factor also affected caregiver QoL through caregiver burden as a mediator indirectly. Poor self-rated health was the strongest factor in the mediating model and might be associated with previous findings in which caregivers, especially those who took care of first-time stroke survivors, experienced more impacts on their physical and mental strains due to their new roles as caregivers [5, 29]. Therefore, these reasons resulted in caregivers’ higher caregiving burden and poor QoL [28, 35].

#### Direct predictors, but no mediating effect on caregiver quality of life

Three predictors related to financial issues had a direct and negative effect on overall caregiver QoL, but no indirect mediating effect existed. The result that family income was not associated with caregiver burden (*F* = 1.20, *p* = 0.31) was similar to the findings of Tang et al. [5]. However, Jeong et al. found that income was a predictor of caregiver QoL and had a mediating effect via caregiver burden [3]. It is possible that the patients recruited in the Jeong et al. study were inpatients in Korea [3], whereas our patients were first-time stroke patients discharged from acute hospitals within 1 year who were living in a rural area in Taiwan. Financial factors might have had a more direct impact on overall caregiver QoL, since the financial QoL subscale was the lowest score reported by our caregivers. Moreover, National Health Insurance in Taiwan merely covers patients’ medical fees for hospitalization and outpatient visits. No comprehensive long-term care policy and no coverage of health expenditures were provided once patients were discharged from hospital during the study period. In the near future, a national compulsory long-term care insurance (LTCI) program in Taiwan will be implemented; this program plans to provide more financial solutions for patients and families for the non-acute stage of illness. Thus, the results from different research might be associated with different caregiver characteristics, patient characteristics, the staging of illness, and health subsidies in different countries.

This study has several limitations. First, our results cannot be generalized to the overall population of stroke survivors, as most of our subjects lived in rural areas, were moderately dependent, and had all been discharged from hospital within a year. Second, this was a cross-sectional study. It remains unknown whether caregivers were able to adapt to caregiving. A follow-up prospective or intervention study to identify changes in the QoL of caregivers is strongly suggested. Third, this study did not include some other significant factors associated with caregiver QoL. Further research needs to explore variables such as social support to explore other potential predictors for QoL of caregivers who were taking care of first-time stroke patients at home.

## Conclusions and implications

Our findings supported our hypothesis, and the financial factors, including lower monthly family income and the spouse paying the patient’s medical fees, had direct negative effects on caregiver QoL without mediation by caregiver burden. Moreover, the caregivers’ poor self-rated health and low educational background directly contributed to the caregivers’ poor QoL and their higher caregiver burden, and these predictors affect caregiver QoL indirectly with caregiver burden as a mediator as well. Therefore, interventions aimed at managing financial issues and alleviating caregiver burden by enhancing caregiver health and decreasing their gaps in stroke-related knowledge and caregiving skill may be effective in promoting caregiver QoL.

To improve caregiver QoL, family caregiver issues should be incorporated into healthcare system designs and policy implementations [36]. The Taiwanese government has promoted long-term care (LTC) policies in three stages since 2008. The first two stages focus on providing patients a home-care service system and community health care. However, the financial relief for caregivers is still limited [37]. In the near future, the third stage of LTC policies, a National compulsory long-term care insurance (LTCI) program, will include an assessment of caregiver strain index (CSI) and provide more medical care services and cash pay out to caregivers. LTCI will remit more caregivers’ financial burden than the first two stages. According to our research and experiences from other countries, we make several suggestions below for policy makers and health providers to promote the QoL of post-stroke family caregivers.

First, regarding caregivers’ financial issues, the following policies may be taken into consideration: (1) Cash benefits, medical benefits or life subsidy; in Germany and the Netherlands, the family receives cash benefits or medical benefits from their LTCI [38]. These cash benefits and contributions to their mandatory pension funds may directly improve caregivers’ financial QoL and encourage caregivers to take care of their family [39]. Additionally, health care insurance provides a subsidy for caregivers on leave or unemployed in Germany [40]. This approach could especially benefit primary caregivers or the major-payer for patients’ medical fees. (2) Expansion of institutionalization: Naomi et al. suggests that institutional care for a patient with a high degree of dependence could cost less than home care for families’ long-term care expansion in a remote area in Japan [41]. This type of care may help stroke patients with high dependency or those living in rural areas when providing subsidy to their institutions and support facilities are needed. (3) Coordinating multiple coverage between health and social policies: the Japanese LTCI scheme covers comprehensive health care and welfare services to relieve the financial burden on family caregivers [39]. LTCI and service delivery in South Korea provides multiple help, such as in-home benefits, facility benefits, family-care cash benefits and hospitalizations attending cash benefits [42]. These multiple financial supports may apply to post-stroke families and include discharge care plans and social and health policies systems as a whole, especially customized for lower-income families to provide appropriate help.

Second, regarding caregivers’ education level and health issues, we recommend providing those caregivers with easy-to-follow care instructions, tailored consultation, and free nursing-care training courses through discharge care plans, workshops or support groups. Additionally, health providers should consider not only patients’ conditions but also caregivers’ health status when providing community health services.

## Abbreviations

ADL: Activities of daily living
BI: Bartel Index
BootLLCI: Bootstrapped 95% CI of lower limit
BootULCI: Bootstrapped 95% CI of upper limit
CI: Confidence interval
CQLI: Caregiver Quality of Life Index
CSI: Caregiver Strain Index
LTC: Long term care
LTCI: Long term care insurance
NG: Nasogastric tube
NTD: New Taiwan Dollar
QoL: Quality of life
SD: Standard deviation
SE: Standard error
SPMSQ: Short Portable Mental Status Questionnaire

## References

[1] Greenwood N, Mackenzie A, Cloud GC, Wilson N (2009). Informal primary carers of stroke survivors living at home-challenges, satisfactions and coping: A systematic review of qualitative studies. Disabil Rehabil.

[2] Yeung EHL, Szeto A, Richardson D, Lai SH, Lim E, Cameron JI (2015). The experiences and needs of Chinese-Canadian stroke survivors and family caregivers as they re-integrate into the community. Health Soc Care Community.

[3] Jeong YG, Jeong YJ, Kim WC, Kim JS (2015). The mediating effect of caregiver burden on the caregivers' quality of life. J Phys Ther Sci.

[4] Nir Z, Greenberger C, Bachner YG (2009). Profile, burden, and quality of life of Israeli stroke survivor caregivers: A longitudinal study. J Neurosci Nurs..

[5] Tang WK, Lau CG, Mok V, Ungvari GS, Wong KS (2011). Burden of Chinese stroke family caregivers: The Hong Kong experience. Arch Phys Med Rehabil.

[6] Vincent-Onabajo G, Ali A, Hamzat T (2013). Quality of life of Nigerian informal caregivers of community-dwelling stroke survivors. J Caring Sci.

[7] Haley WE, Roth DL, Hovater M, Clay OJ (2015). Long-term impact of stroke on family caregiver well-being: A population-based case-control study. Neurology.

[8] Mackenzie A, Greenwood N (2012). Positive experiences of caregiving in stroke: A systematic review. Disabil Rehabil.

[9] Jeong YG, Myong JP, Koo JW (2015). The modifying role of caregiver burden on predictors of quality of life of caregivers of hospitalized chronic stroke patients. Disabil Health J.

[10] Jönsson AC, Lindgren I, Hallström B, Norrving B, Lindgren A (2005). Determinants of quality of life in stroke survivors and their informal caregivers. Stroke.

[11] Ogunlana MO, Dada OO, Oyewo OS, Odole AC, Ogunsan MO (2014). Quality of life and burden of informal caregivers of stroke survivors. HKPJ.

[12] Morimoto T, Schreiner AS, Asano H (2003). Caregiver burden and health-related quality of life among Japanese stroke caregivers. Age Ageing.

[13] MacKinnon DP, Fairchild AJ, Fritz MS (2007). Mediation analysis. Annu Rev Psychol.

[14] McCullagh E, Brigstocke G, Donaldson N, Kalra L (2005). Determinants of caregiving burden and quality of life in caregivers of stroke patients. Stroke.

[15] Mahoney FI, Barthel DW (1965). Functional evaluation: The Barthel index. Md State Med J.

[16] Pfeiffer E (1975). A short portable mental status questionnaire for the assessment of organic brain deficit in elderly patients. J Am Geriatr Soc.

[17] Yen CH, Yeh CJ, Wang CC, Liao WC, Chen SC, Chen CC (2010). Determinants of cognitive impairment over time among the elderly in Taiwan: Results of the national longitudinal study. Arch Gerontol Geriatr.

[18] Robinson B (1983). Validation of a caregiver strain Index. J Gerontol.

[19] Lin LH, Lin CJ, Chen ML (2004). Caregiver burden and related factors between caregivers of post-surgical hospitalized cancer and non-cancer patients: A pilot study. Chang Gung Nursing.

[20] McMillan SC, Mahon M (1994). The impact of hospice services on the quality of life of primary caregiver. Oncol Nurs Forum.

[21] Nijboer C, Triemstra M, Tempelaar R, Sanderman R, van den Bos GA (1999). Determinants of caregiving experiences and mental health of partners of cancer patients. Cancer.

[22] Chen ML, Chu L, Chen HC (2004). Impact of cancer patients' quality of life on that of spouse caregivers. Support Care in Cancer.

[23] Hu LJ (1999). The caregiving burden, depression and quality of life of primary caregivers of metastatic cancer patients receiving home care. Master thesis.

[24] Cohen J (1988). Statistical power analysis for the behavioral sciences.

[25] Hayes AF (2013). Introduction to mediation, moderation, and conditional process analysis.

[26] Preacher KJ, Hayes AF (2008). Asymptotic and resampling strategies for assessing and comparing indirect effects in multiple mediator models. Behav Res Methods.

[27] Yu Y, Hu J, Efird JT, McCoy TP (2013). Social support, coping strategies and health-related quality of life among primary caregivers of stroke survivors in China. J Clin Nurs.

[28] Saban KL, Sherwood PR, DeVon HA, Hynes DM (2010). Measures of psychological stress and physical health in family caregivers of stroke survivors: A literature review. J Neurosci Nurs.

[29] Jeong YJ, Kim WC, Kim YS, Choi KW, Son SY, Jeong YG (2014). The relationship between rehabilitation and changes in depression in stroke patients. J Phys Ther Sci.

[30] Cecil R, Thompson K, Parahoo K, McCaughan E (2013). Towards an understanding of the lives of families affected by stroke: A qualitative study of home carers. J Adv Nurs.

[31] Green TL, King KM (2009). Experiences of male patients and wife-caregivers in the first year post-discharge following minor stroke: A descriptive qualitative study. Int J Nurs Stud.

[32] Tzeng NS, Chang CW, Hsu JY, Chou YC, Chang HA, Kao YC (2015). Caregiver burden for patients with dementia with or without hiring foreign health aides: A cross-sectional study in a northern Taiwan memory clinic. J Med Sci.

[33] Yang X, Hao Y, George SM, Wang L (2012). Factors associated with health-related quality of life among Chinese caregivers of the older adults living in the community: A cross-sectional study. Health Qual Life Outcomes.

[34] Lee HS, Ann CS, Kim MC, Choi JH, Yuk GC (2011). Patient preference for community-based rehabilitation programs after stroke. J Phys Ther Sci.

[35] Bakas T, Clark PC, Kelly-Hayes M, King RB, Lutz BJ, Miller EL (2014). Evidence for stroke family caregiver and dyad interventions: A statement for healthcare professionals from the American Heart Association and American Stroke Association. Stroke.

[36] Levine C, Halper D, Peist A, Gould DA (2010). Bridging troubled waters: Family caregivers, transitions, and long-term care. Health affairs (Project Hope).

[37] Chou YC (2016). Comparison of disability policy models and long-term care policies in Germany and Taiwan: A rehabilitation-oriented perspective. NTU Soc Work Revi.

[38] Liang YU, Hsu MY (2010). A comparison of Long-Term Care Insurance in Germany and the Netherlands. J Nurs.

[39] Chung CC (2012). Lessons from public Long-term Care Insurance in Germany and Japan. J Taiwan Health Care Associ.

[40] Rothgang H (2010). Social insurance for long-term care: An evaluation of German model. Soc Policy Adm.

[41] Naomi A, Shiroiwa T, Fukuda T, Murashima S (2012). Institutional care versus home care for the elderly in a rural area: Cost comparison in rural Japan. Rural Remote Health.

[42] Chen LC (2015). The implementation of Long-term Care Insurance and service delivery in South Korea: A reference for Taiwan. J Long-term Care.

